# Hunner-Type (Classic) Interstitial Cystitis: A Distinct Inflammatory Disorder Characterized by Pancystitis, with Frequent Expansion of Clonal B-Cells and Epithelial Denudation

**DOI:** 10.1371/journal.pone.0143316

**Published:** 2015-11-20

**Authors:** Daichi Maeda, Yoshiyuki Akiyama, Teppei Morikawa, Akiko Kunita, Yasunori Ota, Hiroto Katoh, Aya Niimi, Akira Nomiya, Shumpei Ishikawa, Akiteru Goto, Yasuhiko Igawa, Masashi Fukayama, Yukio Homma

**Affiliations:** 1 Department of Pathology, Graduate School of Medicine, The University of Tokyo, Tokyo, Japan; 2 Department of Cellular and Organ Pathology, Graduate School of Medicine, Akita University, Akita, Japan; 3 Department of Urology, Graduate School of Medicine, The University of Tokyo, Tokyo, Japan; 4 Department of Continence Medicine, Graduate School of Medicine, The University of Tokyo, Tokyo, Japan; 5 Department of Pathology, The Institute of Medical Science, The University of Tokyo, Tokyo, Japan; 6 Department of Genomic Pathology, Medical Research Institute, Tokyo Medical and Dental University, Tokyo, Japan; Oklahoma University Health Sciences Center, UNITED STATES

## Abstract

Interstitial cystitis (IC) is a chronic bladder disease with urinary frequency, bladder discomfort or bladder pain of unknown etiology. Based on cystoscopic findings, patients with IC are classified as either Hunner-type/classic IC (HIC), presenting with a specific Hunner lesion, or non-Hunner-type IC (NHIC), presenting with no Hunner lesion, but post-hydrodistension mucosal bleeding. Inflammatory cell infiltration, composed predominantly of lymphocytes, plasma cells and epithelial denudation, has in the past been documented as a major pathological IC finding. However, the significance of the pathological evaluation of IC, especially with regard to the difference between HIC and NHIC, has been downplayed in recent years. In this study, we performed immunohistochemical quantification of infiltrating T-lymphocytes, B-lymphocytes and plasma cells, and measured the amount of residual epithelium in urinary bladder biopsy specimens taken from patients with HIC and NHIC, and those with no IC, using image analysis software. In addition, *in situ* hybridization of the light chains was performed to examine clonal B-cell expansion. Lymphoplasmacytic infiltration was significantly more severe in HIC specimens than in NHIC specimens (*P* <0.0001). Substantial lymphoplasmacytic inflammation (≥200 cells/mm^2^) was observed in 93% of HIC specimens, whereas only 8% of NHIC specimens were inflamed. Plasmacytic infiltration was more prominent in HIC specimens compared with NHIC and non-IC cystitis specimens (*P* <0.005). Furthermore, expansion of light-chain-restricted B-cells was observed in 31% of cases of HIC. The amount of residual epithelium was decreased in HIC specimens compared with NHIC specimens and non-IC cystitis specimens (*P* <0.0001). These results suggest that NHIC and HIC are distinct pathological entities, with the latter characterized by pancystitis, frequent clonal B-cell expansion and epithelial denudation. An abnormality in the B-cell population may be involved in the pathogenesis of HIC.

## Introduction

Interstitial cystitis (IC) is a chronic disorder characterised clinically by urinary symptoms of frequency, urgency and/or pain in the absence of any identifiable cause, such as infection, obstruction or neoplasia. [1,2] IC diagnosis is based on subjective symptoms combined with cystoscopic findings, and is considered to be a heterogeneous entity with a variety of pathophysiological backgrounds. [2] The etiology of IC is unknown. Although epithelial barrier abnormalities, toxic substances in the urine, and inflammatory, neurogenic or vascular disorders have been suggested as possible mechanisms of IC, none have been conclusive. [3] An autoimmune etiology is also suspected based on female predominance and frequent overlap between IC and other known autoimmune disorders, such as Sjögren’s syndrome, systemic lupus erythematosus, rheumatoid arthritis and ulcerative colitis. [1, 2] Using cystoscopy, IC can be classified into two types: classic or Hunner-type IC (HIC), with Hunner lesions/ulcers (patches of red mucosa exhibiting small vessels radiating to a central pale scar); and non-Hunner-type IC (NHIC), with no identifiable Hunner lesions (HLs) but post-hydrodistension mucosal bleeding. Although some researchers consider HIC and NHIC to be separate entities that differ in terms of clinicopathological features, [3–5] subclassification of IC is not always performed in the clinical context and most surgical pathologists are unaware of the fact that there are two types of IC.

Histologically, stromal inflammation characterised by infiltration of lymphocytes and plasma cells, edema, fibrosis, denudation of mucosal epithelium and detrusor mastocytosis are generally well-documented features of IC. [6, 7] However, most of these findings are considered to be non-specific, chronic inflammatory changes. Moreover, accurate and reproducible assessment of the degree of each histological alteration is quite difficult using conventional evaluation methods measuring semi-quantitative analysis. As a consequence, there has been a trend towards downplaying the histological confirmation of inflammation in patients with IC. In fact, the National Institute of Diabetes and Digestive and Kidney Diseases (NIDDK) criteria do not even require histological assessment for the diagnosis of IC. [8] The authors, DM and TM, who are constantly engaged in pathological assessment of IC specimens in a hospital in which urologists routinely perform bladder biopsies on patients with IC, often see significant variations in the degree of inflammation. Heterogeneity in cases of IC, encompassing a variety of lesions, from non-inflammatory to severely inflamed, is definitely making it difficult to understand the disease. Thus, we recognized the urgent need for precise histological evaluation of IC specimens that could potentially lead to a redefinition of IC.

In the present study, we applied novel image-analysing software to evaluate the extent of inflammation and degree of epithelial denudation in an objective and accurate manner. We paid special attention to the difference between the histology of HIC and that of NHIC, and the difference between the histology of Hunner lesion (HIC-HL) and that of the background bladder mucosa (HIC-BG) in patients with HIC. Furthermore, by comparison of HIC and non-IC cystitis specimens, we tried to clarify whether epithelial denudation occurs specifically in HIC or is a non-specific consequence of chronic inflammation. Last, we tried to investigate the clonality of infiltrating B-cells in IC specimens by detecting light-chain restriction. Evidence with regard to B-cell alteration in IC is scarce. Our goal was to reveal a B-cell alteration that may be associated with the pathogenesis of IC.

## Materials and Methods

### Tissue samples

A total of 93 cold cup biopsy specimens obtained from 66 patients with IC were retrieved from the archives of the Department of Pathology at the University of Tokyo Hospital. This series included 27 consecutive cases of HIC for which biopsy from Hunner lesion and random biopsy from background (non-Hunner lesion) mucosa were performed in 2011, and 39 consecutive cases of NHIC in which one biopsy was randomly taken from the urinary bladder mucosa during 2008–11. The diagnosis of IC was made based on clinical guidelines for IC and hypersensitive bladder syndrome, [9] which requires symptoms, cystoscopic findings and exclusion of diseases that could be confused as IC, by urologists specialising in IC (YH and ANo). Cases were classified as HIC if HLs could be identified under a cystoscope. The rest of the cases revealing no HLs, but demonstrating spotty bleeding (glomerulations) after hydrodistension, were classified as NHIC. Furthermore, the histology of the bladder biopsy specimens taken from non-IC patients during 2009–14 was reviewed. Among them, we selected 26 specimens from 26 patients (15 males and 11 females; mean age 72.7 years), which showed, histologically, roughly the same degree of chronic inflammation as the HIC specimens. We designated them the ‘non-IC cystitis’ group. Of these specimens, seven were bladder biopsies of patients with non-specific chronic cystitis, nine were biopsied from the background non-neoplastic mucosa of patients with bladder cancer and ten were follow-up biopsy specimens from patients with a previous history of bladder cancer.

### Ethical issues

Ethical approval was obtained from the University of Tokyo, Faculty of Medicine, Ethics Committee (Reference Nos 3124 and 2381) and Akita University, Faculty of Medicine, Ethics Committee (Reference No.1247). Written informed consent from the patient was obtained for the use of sample in research. The data were analysed anonymously.

### Clinical survey of patients with IC

We examined the medical records and demographics of patients with IC; the data included sex, age at the time of biopsy, age of onset, O’Leary and Sant’s symptom index and problem index (OSSI/OSPI), cystoscopic findings and the maximum bladder capacity at hydrodistension with a pressure of 80 cmH_2_O at the time of biopsy.

### Histological evaluation of IC biopsy specimens by conventional semi-quantitative (eye-measured) methods

Haematoxylin and eosin (H&E)-stained slides of all the IC cases were reviewed. Initially, the following factors were evaluated semi-quantitatively by eye: degree of stromal inflammation (grade 0: none or minimal inflammatory cell infiltration that can be considered within normal range; grade 1: mild; grade 2: moderate to severe), predominant component of infiltrating inflammatory cells (lymphoplasmacytic inflammation or granulocytic inflammation), ‘plasma cell-rich area’, defined as an area with more than a third of the inflammatory cells as plasma cells (present or absent), lymphoid aggregates/follicles (present or absent), eosinophilic infiltration (present or absent), neutrophilic infiltration (present or absent), full-thickness epithelium (any remaining or completely absent) and degree of epithelial loss/denudation (grade 0/normal: less than a third of the epithelium lost; grade 1/mild: a third to two-thirds of the epithelium lost; grade 2/moderate to severe: more than two-thirds of the epithelium lost).

### Immunohistochemistry and *in situ* hybridization

All the tissue samples were fixed in formalin and embedded in paraffin. Full-thickness tissue sections (4 μm thick) were used for immunohistochemistry (IHC) and *in situ* hybridization (ISH) in all cases. IHC staining and ISH was performed according to standard techniques on a Ventana Benchmark® XT autostainer (Ventana Medical Systems, Tucson, AZ, USA). Appropriate controls were included. We used the antibodies CD3 (1:50, Clone LN10; Novocastra, Newcastle upon Tyne, UK), CD20 (1:100, Clone L26, Dako, Glostrup, Denmark), CD138 (prediluted, Clone B-A38, Nichirei Bioscience, Tokyo, Japan) and cytokeratin (1:100, Clone AE1+AE3, Dako, Glostrup, Denmark) to detect T-lymphocytes, B-lymphocytes, plasma cells and residual epithelium, respectively. We further performed κ- and λ-ISH (Ventana Medical Systems) in HIC specimens and non-IC cystitis specimens.

### Image analysis

**Fig 1:**
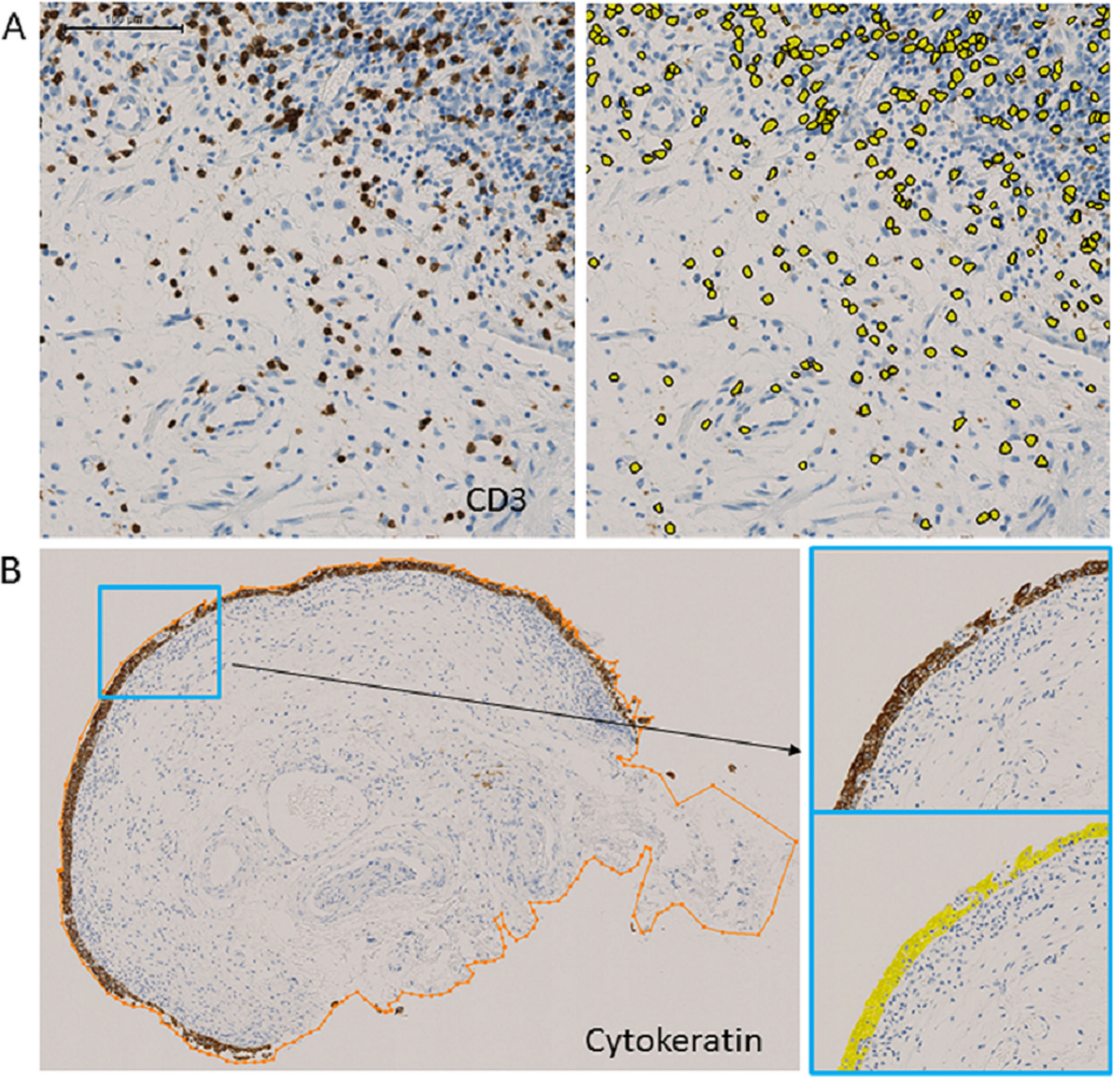
Digital image analysis. (A) Quantification of CD3-positive cells. CD3 immunostaining (left) and identification of CD3-positive cells by image analysis software (right). (B) Evaluation of epithelium/specimen ratio. Measurement of specimen area, circled in orange (left), and identification and measurement of cytokeratin-positive area (right).

Images of immunostained whole slides were digitized using the NanoZoomer Digital Pathology system (Hamamatsu Photonics, Hamamatsu, Japan). For digital quantification, image analysis software (Tissue Studio v.3.5, Definiens AG, Munich, Germany) was used. The numbers of infiltrating T-lymphocytes, B-lymphocytes, and plasma cells were evaluated in the submucosal region, which includes the lamina propria and muscularis propria, if present in the specimen, and their densities were calculated by dividing the number of CD3-, CD20-, and CD138-positive cells by the area (cells/mm^2^). (Fig 1A). For these markers, we performed a manual ROI-nuclei (positive vs. negative) analysis using Tissue Studio software. This mode was the most suitable for detecting the minute nodular staining pattern. After using ‘manual ROI selection’ to circle submucosal areas, the ‘nucleus detection’ module was used with the ‘IHC threshold’ at 0.3 for CD3 and CD20, and 0.5 for CD138, Kappa, and Lambda. The plasma cell ratio was defined as the percentage of CD138-positive cells among the lymphoplasmacytic cells (sum of CD3-, CD20- and CD138-positive cells). The density of κ-ISH-positive cells and λ-ISH-positive cells was calculated in the same manner. Specimens with diffuse background stromal staining for light-chain ISH were excluded from the analysis due to technical difficulties in applying image analysis. We then assessed the clonality of infiltrating B-cells by evaluating the light-chain restriction. In accordance with routine practice for pathological diagnosis, we defined light-chain restriction as an aberrant κ:λ ratio (>5.5 or <0.7), [10] observed in a substantial number of light-chain-positive cells (density: >50 cells/mm^2^). To assess the degree of epithelial denudation or loss, we first quantified the amount of epithelium by measuring the cytokeratin-positive areas. With Tissue Studio software, we performed manual ROI-marker area analysis. First, the ‘manual ROI selection’ module was used to circle the entire tissue specimen. Then, the ‘marker area detection’ module was used with the ‘threshold marker’ set to 0.4. Lastly, we adjusted for the variability in tissue sample size by calculating the proportion of cytokeratin-positive areas per whole tissue sample area (Fig 1B)–the ‘epithelium/specimen ratio (%)’.

### Statistical analysis

Statistical analyses, including Fisher’s exact test, the χ^2^ test for trend, the Mann–Whitney test and Spearman’s rank correlation, were performed using GraphPad PRISM 6.0 software (GraphPad Software, Inc., La Jolla, CA, USA). *P* <0.05 was considered statistically significant.

## Results

### Clinical features of NHIC and HIC

The clinical features of NHIC and HIC are summarized in Table 1. Females predominated in both groups. The onset of NHIC was earlier than that of HIC (*P* = 0.0020), and the age at the time of bladder biopsy was significantly younger in the NHIC group (*P* = 0.0054). Bladder capacity tended to be preserved more in patients with NHIC. However, factors associated with patients’ complaints, such as symptom score (OSSI), problem score (OSPI) and frequency of urination, did not differ between the two groups. None of the IC cases presented with a cystoscopically recognizable tumour or mass at the time of biopsy, and no patient subsequently developed an enlarging mass.

**Table 1:** Clinical features of patients with interstitial cystitis (IC).

Clinical feature	NHIC	HIC	*P* value
Sex			0.0776
Male	12	3	
Female	27	24	
Age at the time of biopsy (years)			0.0054
≤40	11	2	
41–60	7	1	
≥61	21	24	
Age at the onset of disease (years)			0.0020
≤40	12	2	
41–60	13	5	
≥61	14	20	
OSSI			0.9376
0–7	3	5	
8–14	25	11	
≥15	11	10	
OSPI			0.5734
0–7	7	4	
8–14	24	15	
≥15	8	7	
Frequency of urination (/day)			0.4894
0–10	13	5	
11–20	19	17	
≥21	7	4	
Maximum bladder capacity (mL)			0.0064
≤400	3	8	
401–800	29	17	
>800	7	1	

HIC, Hunner-type/classic IC; NHIC, non-Hunner-type IC; OSPI, O’Leary and Sant’s problem index; OSSI, O’Leary and Sant’s symptom index.

### Histological comparison between NHIC and HIC specimens by conventional semi-quantitative methods

**Fig 2:**
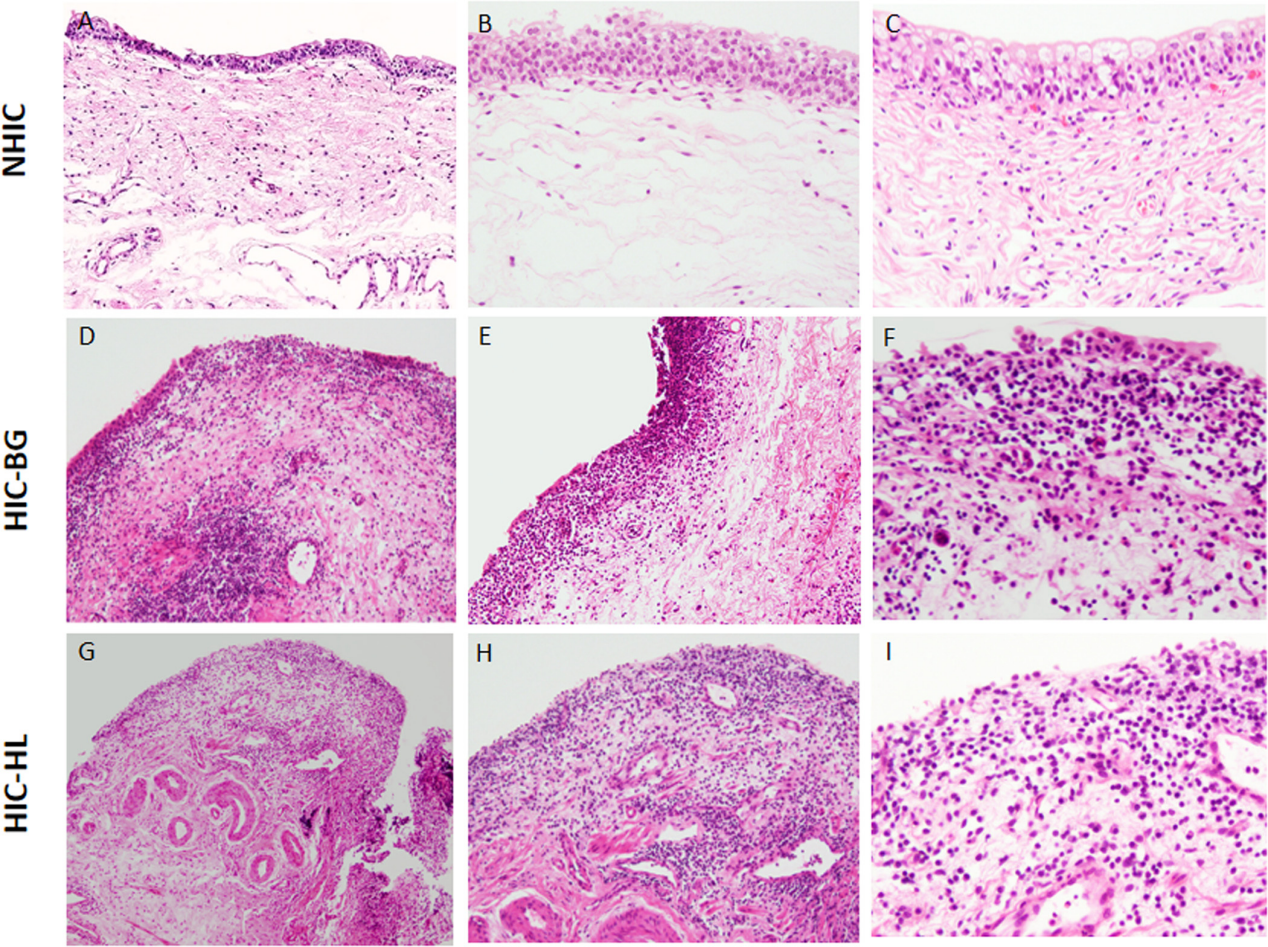
Representative histology of non-Hunner-type interstitial cystitis (NHIC) biopsy specimen. (A) low-power view reveals unremarkable bladder mucosa; (B) overlying epithelium is well preserved; no stromal inflammation is seen; (C) lymphocytes are only occasionally found in the lamina propria. (D–F) Representative histology of the HIC biopsy specimen taken from background (non-Hunner lesion) mucosa (HIC-BG): (D) diffuse inflammatory cell infiltration with focal aggregate of lymphocytes present in the subepithelial layer; (E) dense subepithelial inflammation observed in a linear pattern; (F) inflammatory cells predominantly composed of lymphocytes and plasma cells; epithelium is irregularly denuded. (G–I) Representative histology of the Hunner lesions of patients with HIC: (G) low-power view; (H) subepithelial layer diffusely inflamed; (I) epithelium completely denuded and numerous plasma cells found in the underlying stroma.

The results of morphological evaluation of IC specimens using conventional methods, semi-quantitative analysis, are summarized in Table 2. Most HIC specimens showed at least some inflammatory change and the epithelium was frequently denuded. On the other hand, a vast majority of NHIC specimens were not inflamed and the epithelium tended to be preserved. In all cases with stromal inflammation (grades 1 and 2), the predominant component of the infiltrating inflammatory cells was lymphoplasmacytic cells, which outnumbered the granulocytes (eosinophils and neutrophils). In HIC cases, HLs and the BG were similarly inflamed. The extent of epithelial denudation/loss looked more severe in HIC-HL than in HIC-BG (*P* <0.0001). Fig 2 illustrates the representative histology of HIC and NHIC specimens.

**Table 2:** Semi-quantitative analysis of pathological features of interstitial cystitis (IC) biopsy specimens.

Pathological feature	NHIC	HIC-BG	HIC-HL	*P* value
	*n* = 39	*n* = 27	*n* = 27	
				NHIC vs HIC-BG: <0.0001
Subepithelial inflammation				NHIC vs HIC-HL: <0.0001
				HIC-BG vs HIC-HL: 0.0975
Grade 0	37	8	3	
Grade 1	1	11	12	
Grade 2	1	8	12	
				NHIC vs HIC-BG: 0.0025
Lymphoid aggregate/follicle				NHIC vs HIC-HL: <0.0001
				HIC-BG vs HIC-HL: 0.0542
Absent	38	19	11	
Present	1	8	16	
				NHIC vs HIC-BG: 0.0003
Plasma cell-rich area				NHIC vs HIC-HL: <0.0001
				HIC-BG vs HIC-HL: 0.7822
Absent	38	17	15	
Present	1	10	12	
				NHIC vs HIC-BG: 0.0064
Neutrophilic infiltration				NHIC vs HIC-HL: <0.0001
				HIC-BG vs HIC-HL: 0.2542
Absent	38	20	15	
Present	1	7	12	
				NHIC vs HIC-BG: 0.0064
Eosinophilic infiltration				NHIC vs HIC-HL: <0.000
				HIC-BG vs HIC-HL: 0.1480
Absent	37	12	6	
Present	2	15	21	
				NHIC vs HIC-BG: <0.0001
Full-thickness epithelium				NHIC vs HIC-HL: <0.0001
				HIC-BG vs HIC-HL: 0.4142
Present	38	16	12	
Absent	1	11	15	
				NHIC vs HIC-BG: <0.0001
Epithelial denudation/loss				NHIC vs HIC-HL: <0.0001
				HIC-BG vs HIC-HL: 0.0010
Grade 0	33	15	5	
Grade 1	5	7	6	
Grade 2	1	5	16	

BG, background mucosa; HIC, Hunner-type/classic IC; HL, Hunner lesion; NHIC, non-Hunner-type IC.

Although these data give an overview of the differences between HIC and NHIC specimens, accurate grading of inflammation and epithelial denudation/loss was often difficult by eye. As no bladder mucosa, even of those unaffected by urinary disorder, is completely devoid of inflammatory cells, the definition of ‘stromal inflammation: grade 0’ was quite arbitrary. Grading of epithelial denudation/loss was also somewhat ambiguous, because focal artificial epithelial loss occurs frequently in any kind of bladder biopsy specimens. Furthermore, in some cases with severe inflammation, location of the mucosa could not be appreciated readily. These technical limitations prompted us to obtain more objective and quantitative data using image analysis software.

### Quantification of lymphoplasmacytic infiltration and residual epithelium in NHIC, HIC and non-IC cystitis specimens by image analysis

**Fig 3:**
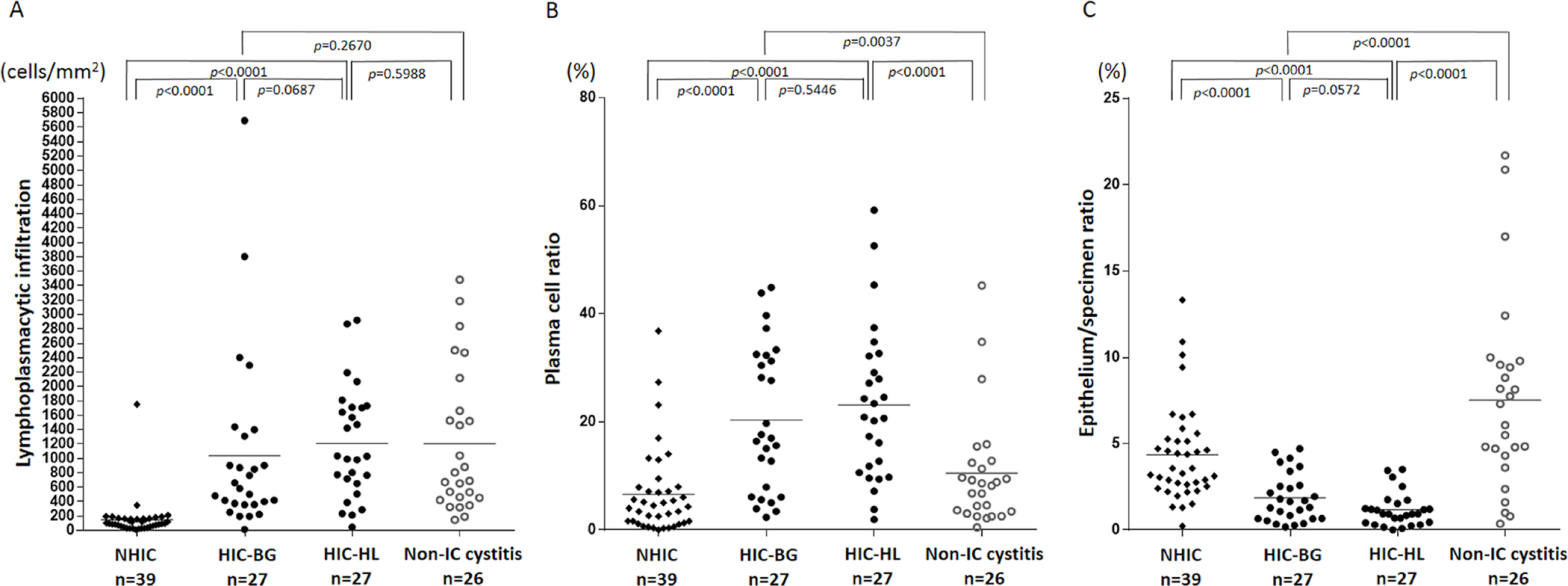
Evaluation of inflammatory cell infiltration and residual epithelium by image analysis software. (A) Lymphoplasmacytic infiltration in non-Hunner-type interstitial cystitis (NHIC), HIC-BG (background mucosa), HIC-HL (Hunner lesion) and non-IC cystitis specimens. Lateral bars indicate the means. Lymphoplasmacytic infiltration was much more severe in HIC-BG and HIC-HL than NHIC. The number of infiltrating mononuclear cells in NHIC specimens was very few (<200 cells/mm^2^) in most cases. The non-IC cystitis group showed a similar degree of mononuclear cell inflammation to that of HIC-BG and HIC-HL. (B) Plasma cell ratio in NHIC, HIC-BG, HIC-HL and non-IC cystitis specimens. This was significantly higher in HIC-HL and HIC-BG than in NHIC and non-IC cystitis. (C) Epithelium/specimen ratio in NHIC, HIC-BG, HIC-HL and non-IC cystitis specimens. Lateral bars indicate the means. The epithelium/specimen ratio is significantly lower in HIC specimens compared with NHIC and non-IC cystitis specimens (*P* <0.0001).

**Fig 4:**
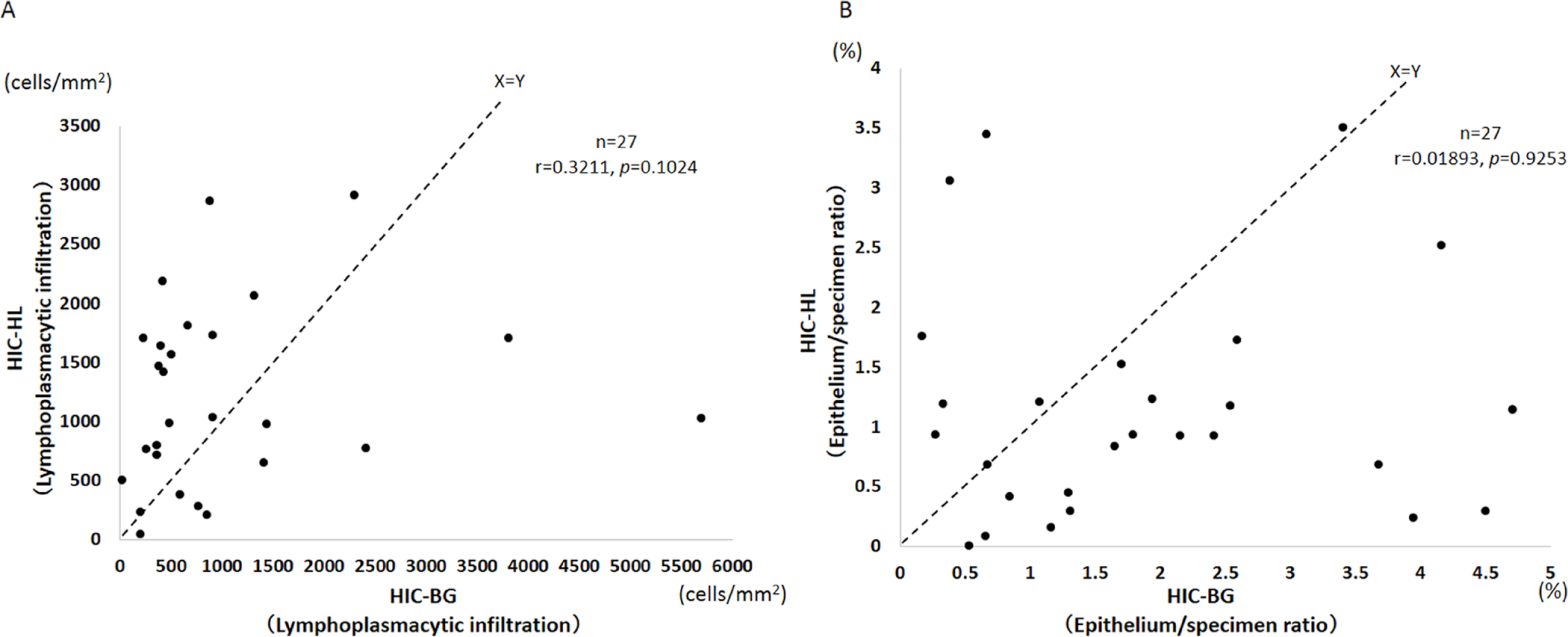
Inflammation and epithelial denudation in HIC cases. (A) Each case of Hunner-type/classic IC (HIC) was plotted for lymphoplasmacytic infiltration in Hunner lesion (HIC-HL) and background mucosa (HIC-BG) (*n* = 27). There was no significant correlation between the degree of inflammation in HIC-HL and that in HIC-BG. In 18 of 27 cases, lymphoplasmacytic infiltration was denser in HLs than in the BG. (B) Each case of HIC was plotted for the epithelium/specimen ratio of HIC-HL and HIC-BG (*n* = 27). There was no significant correlation between the degree of epithelial loss in HIC-HL and that in HIC-BG. In 19 of 27 cases, the epithelium/specimen ratio was lower in HLs than in the BG.

The result of quantitative image analysis of lymphoplasmacytic infiltration is demonstrated in Fig 3A. The density of lymphoplasmacytic infiltration was much higher in HIC-BG and HIC-HL specimens than in NHIC specimens (*P* <0.0001), and HIC-HL specimens tended to contain a larger number of lymphoplasmacytic cells than HIC-BG specimens. However, this difference was not statistically significant (*P* = 0.0687). The non-IC cystitis group showed a similar degree of lymphoplasmacytic inflammation to that of HIC-BG and HIC-HL specimens. The plasma cell ratio was significantly higher in both HIC-BG and HIC-HL than in NHIC and non-IC cystitis specimens (Fig 3B). Comparison of the lymphoplasmacytic infiltration in the HLs and in the BG of each HIC case revealed stronger inflammation in the HLs in 18 of 27 cases. No significant correlation was observed between the degree of lymphoplasmacytic infiltration in the HLs and that in the BG of each case (Fig 4A).

With regard to the degree of inflammation, quantitative image analysis correlated well with semi-quantitative grading (Table 3 and S1 Fig). Lymphoplasmacytic infiltration >200 cells/mm^2^ was observed in all specimens with inflammation grades 1 and 2, whereas lymphoplasmacytic infiltration was <200/mm^2^ in >80% of grade 0 specimens. Based on this finding, we set a cut-off line at 200 lymphoplasmacytic cells/mm^2^, to reclassify IC specimens into ‘normal (no significant inflammation)’ and ‘inflamed’. By applying this criterion, which is stricter than assessing grade 0 versus grade 1 and 2 semi-quantitatively, 50/54 (92.6%) HIC specimens were classified as ‘inflamed’. There was only one HIC case that revealed no significant inflammation in both HLs and the BG. On the other hand, 36/39 (92.3%) NHIC specimens were reclassified as ‘normal’ (no significant inflammation).

**Table 3:** Correlation between lymphoplasmacytic infiltration measured by image analysis and semi-quantitative inflammation grading in interstitial cystitis (IC) biopsy specimens.

	Lymphoplasmacytic infiltration measured by image analysis (cells/mm^2^)					
	<200		200–1000		>1000	
	NHIC	HIC	NHIC	HIC	NHIC	HIC
Ssemi-quantitative inflammation grading Grade 0 (normal)	36	4	1	7	0	0
Ssemi-quantitative inflammation grading Grade 1 (mild)	0	0	1	16	0	7
Ssemi-quantitative inflammation grading Grade 2 (moderate to severe)	0	0	0	6	1	14

HIC, Hunner-type/classic IC; NHIC, non-Hunner-type IC.

The results of the analysis of the epithelium/specimen ratio in IC specimens and non-IC cystitis specimens are summarized in Fig 3C. The epithelium/specimen ratio was much lower in HIC specimens than in NHIC and non-IC cystitis specimens (*P* <0.0001). Epithelial loss tended to be more prominent in HIC-HL than in HIC-BG specimens, but the difference was not statistically significant (*P* = 0.0572). We further compared epithelium/specimen ratios for HLs and the BG in each case of HIC, and showed that epithelial loss/denudation was more severe in HLs than in the BG in 19 of 27 cases (Fig 4B).

Lastly, we compared HIC and cystitis specimens taken from cancer cases (a subgroup of non-IC cystitis). The result is shown in S2 Fig. Even in this subgroup analysis, the plasma cell-rich feature and epithelial denudation were more prominent in HIC.

### Correlation analysis between inflammation and epithelial loss in HIC specimens

**Fig 5:**
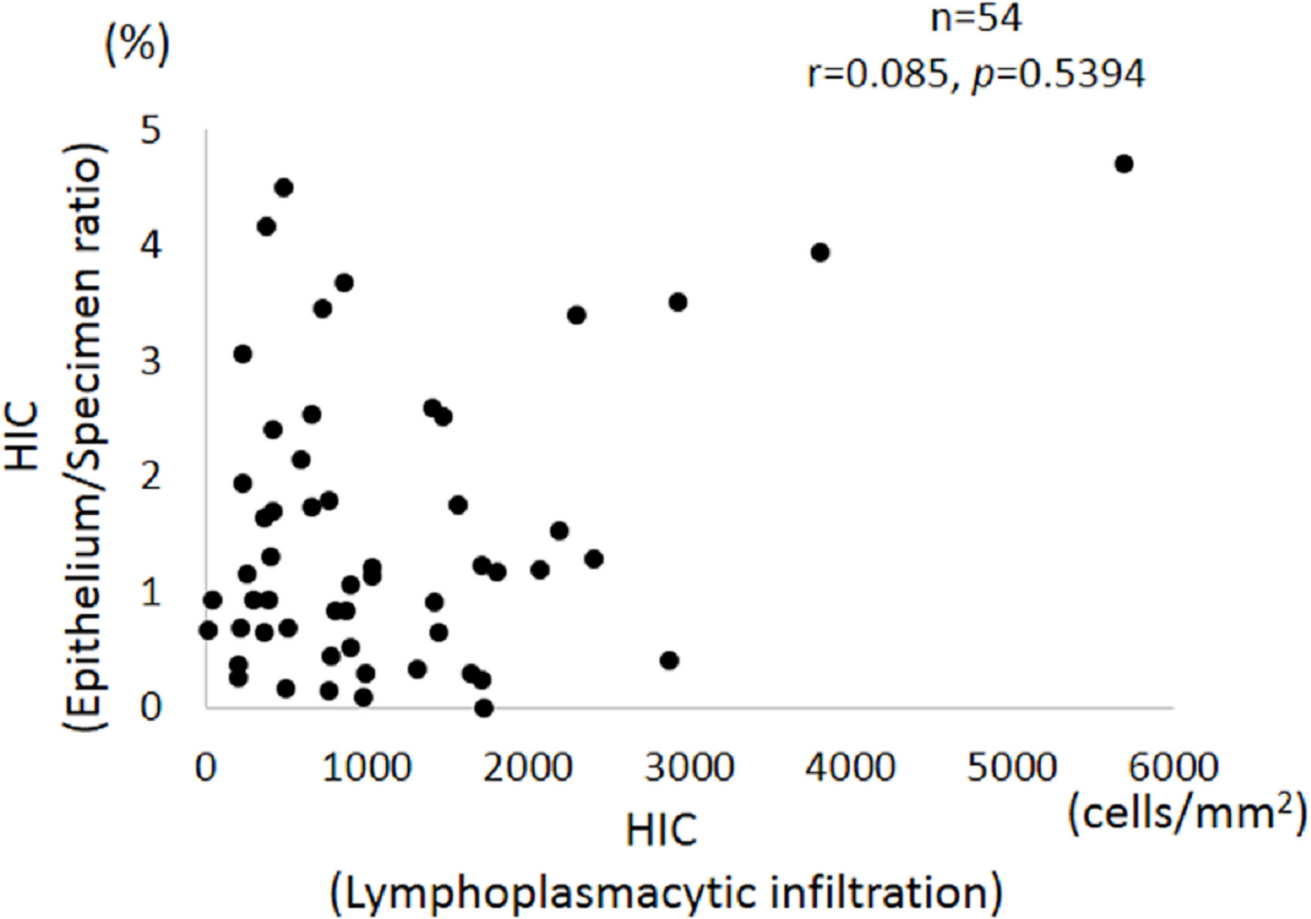
Correlation between lymphoplasmacytic infiltration and epithelium/specimen ratio in Hunner-type/classic IC specimens. Degree of lymphoplasmacytic infiltration did not correlate with the amount of residual epithelium.

All 54 HIC specimens, which included both biopsies from HLs and the BG, were assessed for correlation between the degree of lymphoplasmacytic infiltration and the epithelium/specimen ratio (Fig 5). There was no significant correlation.

### Light-chain restriction in HIC specimens

**Fig 6:**
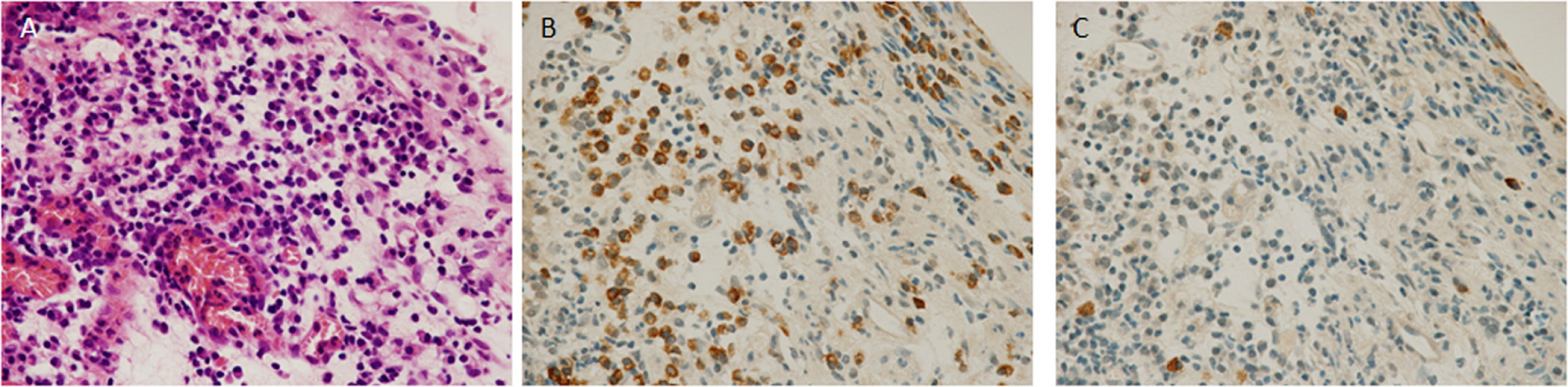
Hunner-type/classic IC (HIC) specimen with expansion of light chain-restricted B cells (κ >>> λ). (A) A dense plasmacytic infiltration is observed in the subepithelial stroma. (B) *In situ* hybridization for the κ chain reveals numerous κ-positive cells. (C) *In situ* hybridization for the λ chain reveals only a few λ-positive cells.

In 31% (8/26) of evaluable HIC cases, light-chain restriction was observed in at least one of the biopsies (Fig 6 and Table 4). In total, 46 HIC biopsy specimens were evaluated, and expansion of light-chain-restricted B-cells was observed in 9. Five specimens were restricted to the κ side and the remainder were restricted to the λ side. The representative histological features of HIC specimens with light-chain restriction are shown in Fig 6. The frequency of light-chain restriction did not differ significantly between HLs (3/21) and the BG (6/25). None of the non-IC cystitis specimens showed expansion of light-chain-restricted B-cells (0/23).

**Table 4:** Light-chain restriction in cases of Hunner-type interstitial cystitis (HIC).

	HIC	
Case	BG	HLs
1	+(κ)	–
2	–	–
3	–	–
4	–	–
5	–	–
6	–	NE
7	–	–
8	–	–
9	–	–
10	–	–
11	–	NE
12	+(κ)	–
13	+(λ)	–
14	–	+(κ)
15	–	NE
16	–	–
17	–	+(κ)
18	+(λ)	+(λ)
19	NE	–
20	–	–
21	+(λ)	NE
22	–	–
23	+(κ)	–
24	–	NE
25	NE	NE
26	–	–
27	–	–

BG, background mucosa; HIC, Hunner-type/classic IC; HL, Hunner lesion; IC, interstitial cystitis; NE, not evaluable due to background staining.

### Clinicopathological analysis of HIC cases

We analyzed the correlation between histological factors including the epithelium/specimen ratio and density of infiltrating T-lymphocytes, B-lymphocytes, plasma cells and their sum (lymphoplasmacytic cells in total), and clinical factors including patient’s age, age of onset, OSSI/OSPI, frequency of urination and maximum bladder capacity at hydrodistension in the HIC cases. As a result, we found no significant correlation between any of the factors assessed.

## Discussion

The first documentation of IC dates back to 1907 when Nitze called it ‘cystitis parenchymatosa’. [11] Hunner later described a characteristic endoscopic lesion (Hunner lesion/ulcer), referring to this disease as ‘a rare type of bladder ulcer in women’. [12] Since these initial reports, urologists have recognized that, in a substantial number of patients with IC symptoms, no HLs, but only ‘glomerulations’ or submucosal bleeding after bladder hydrodistension is observed. [13] Then, broader concepts such as ‘painful bladder syndrome (PBS)’ [14] and ‘bladder pain syndrome (BPS)’, [15] the diagnoses of which are made solely on symptoms, were introduced to redefine IC. Consequently, what is now called PBS/IC or BPS/IC is quite heterogeneous. We, as pathologists, believe that it is essential to classify IC according to the presence or absence of histologically confirmed inflammation, for better understanding of the disease itself, to investigate the pathogenesis of IC in an appropriate manner and to seek for specific treatment options. Histopathologically, lymphoplasmacytic infiltration, edema and fibrosis of the stroma, detrusor mastocytosis and denudation of the mucosal epithelium are well-recognized features of IC. [5–7, 16] Most of these morphological descriptions of IC specimens date back to the 1980s and 1990s. Despite recent progress regarding the neurobiological aspects of the disease,[17–19] the significance of the histological assessment has tended to be downplayed. Consequently, some now consider that many of the pathological features listed above are non-specific. [20, 21]

In the present study, we have clearly shown, with the aid of IHC and image analysis software, that there are two subsets of IC: HIC which is an inflammatory disorder that almost always presents with cystoscopically identifiable HLs, and NHIC which literally shows no evidence of inflammation under the microscope. This observation is consistent with previous reports by Scandinavian researchers who have insisted on distinguishing NHIC and HIC. [3–6, 22] The difference in the degree of lymphoplasmacytic infiltration between NHIC and HIC in our series is so striking that it is difficult to comprehend NHIC as merely a mild form of HIC. Furthermore, the significant difference in plasma cell ratios supports the distinct nature of the two types. The present study suggests that cystoscopic identification of HLs is quite useful in screening IC cases with an underlying inflammatory process. However, accurate detection of HLs may often be difficult because not all urologists specialize in IC. Thus, bladder biopsies and subsequent histological assessment are necessary to confirm inflammation. This idea is in line with the current concept of European criteria that attempt to classify PBS/IC based on both cystoscopic and histological findings. Most HIC cases in our series correspond to the European Society for the Study of IC/PBS (ESSIC) classification 3C (HLs: present; histological evidence of inflammation: present), [14] and we postulate that this subset should be regarded as ‘interstitial cystitis’ in a narrow sense.

As expected, significant loss of the epithelium was observed in HIC specimens. It is interesting that the epithelium of non-IC cystitis specimens was well preserved despite the presence of inflammatory cell infiltration. Therefore, we concluded that epithelial denudation is a specific alteration occurring in the urinary bladder of patients with HIC, and that it does not occur as a consequence of general chronic inflammation. We can simply hypothesize that epithelial loss is the primary cause of HIC and stromal inflammation is a subsequent event. However, there is also a possibility that some special inflammatory process takes place in HIC that could lead to epithelial denudation. At this point, it is not easy to draw any conclusions, but the relationship between the epithelium and inflammation is definitely a subject for future IC studies.

The histological features of HLs have not been clearly defined. In the present series of 27 HIC cases, lymphoplasmacytic inflammation and epithelial denudation were observed in a diffuse manner, regardless of HLs or the BG. Although the degree of lymphoplasmacytic inflammation and epithelial denudation tended to be more severe in HLs than in the BG, the difference was not statistically significant. As the histological factors assessed in this study are limited to lymphoplasmacytic cells and epithelium, other factors such as vascular abnormality and stromal fibrosis may well play an important role in the development of HLs.

Detection of a light-chain-restricted, clonal B-cell population is a novel finding in the field of IC research. In the past, abundance of infiltrating plasma cells, [7, 23, 24] and the increased expression of B-cell-associated genes [25, 26] in IC specimens have been reported. But, no study had assessed the clonality of the infiltrating B-cells. To our surprise, light-chain restriction, direct evidence of clonal B-cell expansion, was observed in >30% of HIC cases in the present study. In most of these cases, light-chain restriction was observed in only one of the two biopsies performed. Therefore, we speculate that clonal B-cell expansion in HIC is usually a focal event not involving the whole bladder. From a pathologically diagnostic point of view, it is extremely difficult to rule out the possibility of an early, minute, mucosa-associated lymphoid tissue (MALT) lymphoma in these HIC biopsy specimens with light-chain restriction. Currently, there are no epidemiological data to suggest an association between IC and malignant lymphoma, and our clinical experience indicates that the focal clonal B-cell expansion in HIC does not readily progress to overt lymphoma.

In general, clonal expansion of B-cells is considered to be a consequence of local immune response and selection of a specific clone of B-cells. It has been shown to occur in a variety of inflammatory diseases, especially in association with autoimmunity. [27] Expansion of monoclonal B-cell populations and the subsequent development of MALT-type lymphoma is a well-known phenomenon in the salivary glands of patients with Sjögren’s syndrome. [28] Recent analyses of the human B-cell receptor repertoire have also shown clonal B-cell expansion in the synovium of patients with rheumatoid arthritis, [29] in the liver tissue of patients with IgG4-related cholangitis [30] and in other chronic inflammatory disorders. [31] Besides the autoimmune process, bacterial infections such as *Helicobacter pylori* gastritis or viral infections such as Epstein–Barr virus infection can induce clonal B-cell expansion. [32] It is of great interest to discover if clonal B-cell expansion is the primary cause of HIC or the consequence of chronic bladder inflammation, whether it occurs in an autoimmune process or infectious disease. Further studies are definitely needed to reveal the biological significance of a B-cell abnormality in HIC.

Last, we found no significant correlation between histopathological factors and clinical factors such as symptom score and bladder volume in HIC cases. It is controversial whether histology correlates with the severity of disease in IC cases.[16, 33–36] Thus, the utility of histological evaluation in IC cases is in practice limited to the distinction between NHIC and HIC. It is difficult to predict the severity of the IC symptoms on the basis of bladder biopsy histology.

In conclusion, HIC is a distinct inflammatory disorder characterized by pancystitis with an increase in plasma cells and frequent expansion of clonal B-cells, and epithelial denudation. A B-cell population abnormality may be involved in the pathogenesis of HIC.

## Supporting Information

S1 Fig Correlation between grading of inflammation semi-quantitative analysis and lymphoplasmacytic infiltration evaluated by image analysis software in interstitial cystitis (IC) specimens. The lateral bars indicate means. Strong correlation was observed between the results of image analysis and grading semi-quantitative analysis (r = 0.853, *P* <0.0001).
(TIF)

S2 Fig Evaluation of inflammatory cell infiltration and residual epithelium in HIC and non-IC cancer cases. (A) Lymphoplasmacytic infiltration (B) Plasma cell ratio (C) Epithelium/specimen ratio.
(TIF)
