# Supplementary figures and images for: Hunner-Type (Classic) Interstitial Cystitis: A Distinct Inflammatory Disorder Characterized by Pancystitis, with Frequent Expansion of Clonal B-Cells and Epithelial Denudation
Source: PLoS One. 2015 Nov 20;10(11):e0143316. doi: 10.1371/journal.pone.0143316 (PMC4654580; doi:10.1371/journal.pone.0143316)

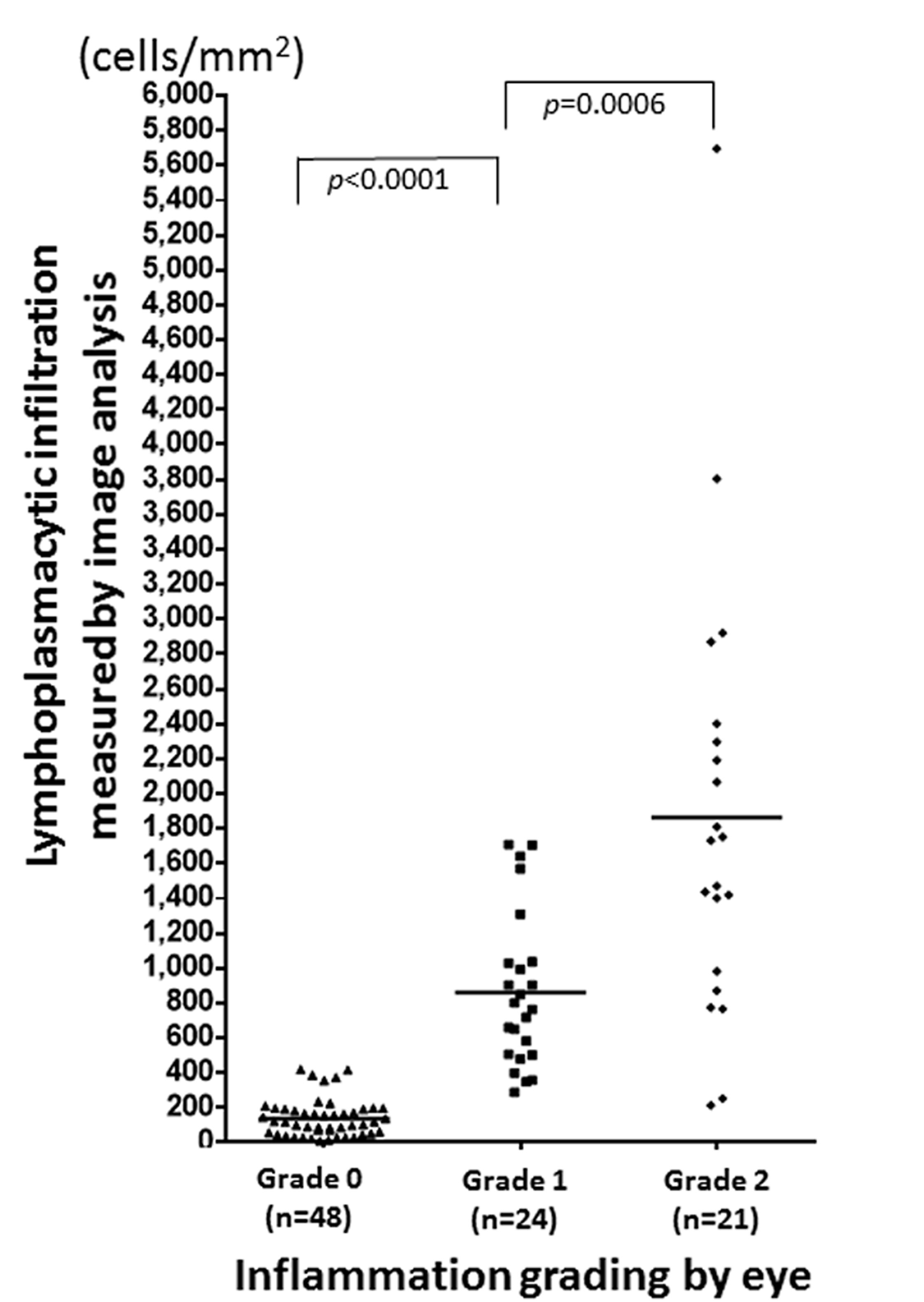

Supplement: S1 Fig — The lateral bars indicate means. Strong correlation was observed between the results of image analysis and grading semi-quantitative analysis (r = 0.853, P <0.0001). (TIF) [file pone.0143316.s001.tif]

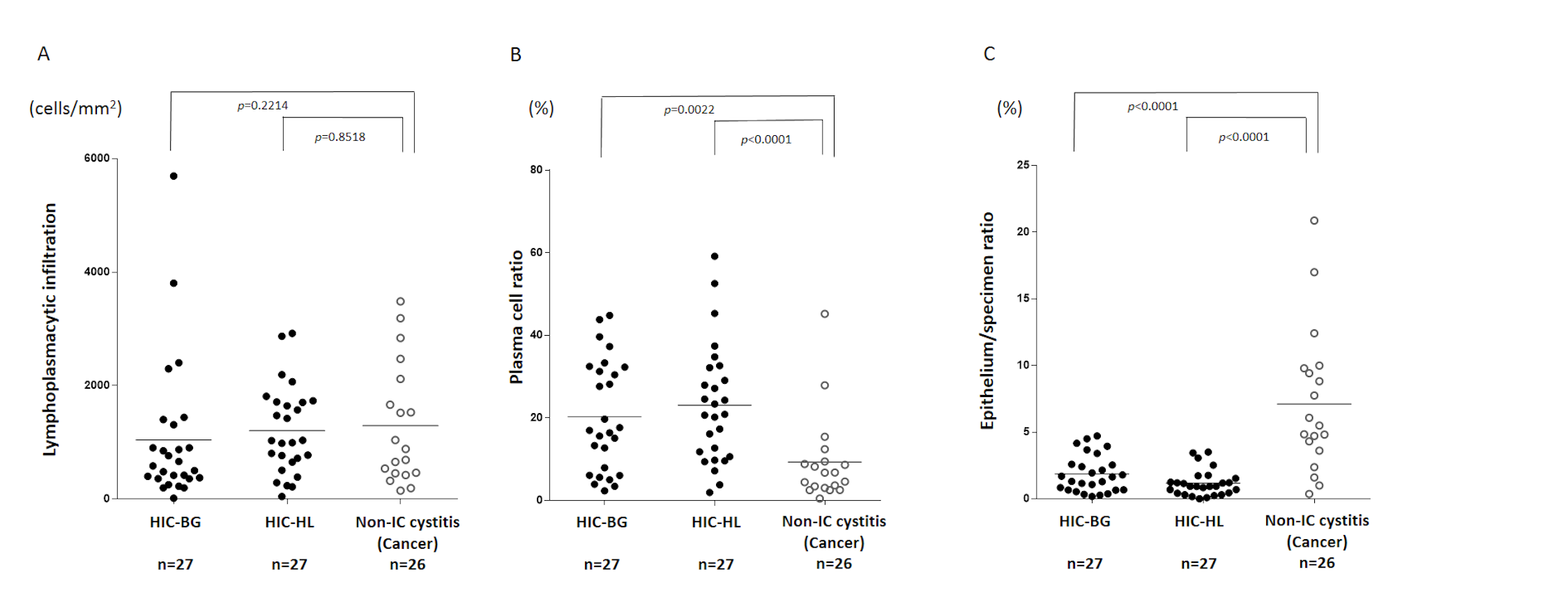

Supplement: S2 Fig — (A) Lymphoplasmacytic infiltration (B) Plasma cell ratio (C) Epithelium/specimen ratio. (TIF) [file pone.0143316.s002.tif]
